# Deep Learning-Based Diagnosis of Peripheral Artery Disease via Continuous Property-Adversarial Regularization: Preliminary in Silico Study

**DOI:** 10.1109/access.2021.3112678

**Published:** 2021-09-14

**Authors:** SOOHO KIM, JIN-OH HAHN, BYENG DONG YOUN

**Affiliations:** 1 Department of Mechanical and Aerospace Engineering, Seoul National University, Seoul 08826, South Korea; 2 OnePredict Inc., Seoul 06160, South Korea; 3 Department of Mechanical Engineering, University of Maryland, College Park, MD 20742, USA; 4 Institute of Advanced Machines and Design, Seoul National University, Seoul 08826, South Korea

**Keywords:** Deep learning, continuous domain-adversarial learning, peripheral artery disease, cardiovascular disease, arterial pulse wave analysis, convolutional neural network

## Abstract

This paper presents a novel deep learning-based arterial pulse wave analysis (PWA) approach to diagnosis of peripheral artery occlusive disease (PAD). Naïve application of deep learning to PAD diagnosis can be hampered by the fact that securing a large amount of longitudinal dataset encompassing diverse PAD severity as well as anatomical and physiological variability presents formidable challenge. Training of a deep neural network (DNN) to a small training dataset raises the risk of overfitting the PAD diagnosis algorithm only to the individuals in the training dataset while deteriorating its ability to generalize also to other individuals who may exhibit a large variability in anatomical and physiological characteristics beyond the training dataset. To overcome these obstacles, we propose a continuous property-adversarial regularization (CPAR) approach to robust generalization of a DNN against scarce datasets. Our approach fosters the exploitation of latent features that can facilitate the intended task independently of confounding property-induced disturbances. by regularizing the extraction of disturbance-dependent latent features in the network’s feature extraction layer. By training and testing a deep convolutional neural network (CNN) for PAD diagnosis using scarce virtual datasets, we illustrated that the CNN trained by our approach was superior to a conventionally trained CNN in detecting and assessing the severity of PAD against disturbances originating from diversity in the patients’ height and arterial stiffness: when trained with one-time pulse wave signal measurement at ankle and brachial arteries in a small number of patients, our approach achieved detection accuracy of >90% and severity assessment of 0.83 in r^2^ value, which were >15% and >40% improvement over conventional approach without CPAR. In addition, we ascertained the advantage of our approach in efficient training and robust generalization of DNN by contrasting it to multi-task learning which promotes the exploitation (as opposed to regularization in CPAR) of disturbance-dependent latent features in fulfilling the intended tasks.

## I. INTRODUCTION

Peripheral artery occlusive disease (PAD) is a highly prevalent vascular disease entailing high morbidity and mortality risks. The United States and the world are estimated to have >8 million (2000 [1]) and >200 million (in 2010 [2]) patients with PAD. In addition, PAD may become even more prevalent with societal aging. Regardless, PAD is underdiagnosed with low primary care awareness [3].

PAD diagnosis in clinical settings requires imaging-based techniques [4]–[6], which involve invasive procedures and/or expensive equipment. Hence, these techniques are not suited to high-throughput PAD screening and surveillance. On the other hand, the ankle-brachial index (ABI), a widely employed technique for PAD screening, has been criticized for its poor accuracy and robustness in diagnosing PAD [7].

The analysis of arterial pulse wave signals (called here-after the pulse wave analysis (PWA)) can play a pivotal role in PAD diagnosis. Indeed, PAD alters the morphology (i.e., shape) of arterial pulse wave signals (e.g., arterial blood pressure (BP) signals) by affecting the pulse wave propagation and reflection characteristics in the arteries. Hence, PWA has the potential to outperform techniques built upon discrete fiducial points (i.e., features) in the arterial pulse wave signals (e.g., ABI) by allowing the exploitation of the arterial pulse wave signals in their entirety [8]–[11]. A practical advantage of PWA is that it may be relevant to affordable and high-throughput PAD screening and surveillance with convenient arterial pulse wave signals measured at the extremity locations (e.g., arm and ankle).

The mainstream of existing work on PWA has resorted to empirical feature selection. Hence, PWA may be combined with modern deep learning (DL) to leverage its ability for automatic feature selection [11]. Yet, an obvious challenge in incorporating DL into PWA is dataset limitation constraint: naïve application of DL to PWA-based PAD diagnosis requires massive longitudinal datasets associated with PAD progression in time, which preferably also encompass diverse anatomical and physiological characteristics as well as disease severity levels. Yet in reality, only scarce (and possibly non-longitudinal) dataset from a small number of patients associated with limited anatomical and physiological characteristics may be available, which may pose formidable challenges in training a deep neural network (DNN) for PAD diagnosis which is generalizable to a wide range of anatomical and physiological characteristics. In fact, the morphology of the arterial pulse wave signals depends not only on PAD but also on the anatomical and physiological characteristics of a patient. For example, mechanical properties of the arterial wall and height of the patient can alter the shape of arterial pulse wave signals by affecting the way forward and backward traveling BP waves are superposed – mechanical properties (e.g., arterial stiffness, diameter, and thickness) by altering pulse wave velocity (PWV) and height by altering the timings of superposition even for a given PWV [12]. Hence, if a DNN is trained using scarce dataset from a small number of patients (who possibly encompass only a narrow range of anatomical and physiological characteristics), the resulting PAD diagnosis algorithm may be overfitted only to the individuals in the training dataset, while its ability to generalize to other individuals (who may exhibit a large variability in anatomical and physiological characteristics beyond the training dataset) may be deteriorated.

To overcome these obstacles, we propose a continuous property-adversarial regularization (CPAR) approach to robust generalization of a DNN against scarce datasets. Inspired by domain-adversarial learning [13], we extend the conventional discrete domain-adversarial regularization to enable regularization and generalization of a DNN across multiple continuous properties by reformulating the anti-domain classification formalism used in discrete domain-adversarial regularization to a novel anti-property regression formalism applicable to multiple continuous properties. In this way, our approach fosters the exploitation of latent features that can facilitate the intended task independently of confounding property-induced disturbances, by regularizing the extraction of disturbance-dependent latent features in the network’s feature extraction layer. By training and testing a deep convolutional neural network (CNN) for PAD diagnosis using scarce virtual datasets, we illustrated that the CNN trained by CPAR was superior to a conventionally trained CNN in detecting PAD and assessing its severity against disturbances originating from diversity in the patients’ height and arterial stiffness.

This paper is organized as follows. Section II covers the details of the CPAR approach and how it was applied to develop and test a CNN-based PAD diagnosis algorithm. Section III presents results, and Section IV provides discussion of the results. Section V concludes the paper with future directions.

## II. METHODS

In this section, we first present the CPAR approach to robust generalization of a DNN against continuous property-induced disturbances. Then, we apply the CPAR approach to PAD diagnosis via CNN-based PWA, in order to efficiently train a CNN using relatively scarce dataset from a small number of patients so that it can robustly generalize to broad patient cohort with wide anatomical and physiological variability.

### A. CONTINUOUS DOMAIN-ADVERSARIAL REGULARIZATION

In circumstances in which a DNN is trained using a scarce dataset consisting of only a small number of samples, the DNN may be overfitted to the training dataset and thus exhibit poor performance when applied to an unseen dataset whose samples are associated with properties not covered by those in the training dataset. Two alternative approaches may be employed to mitigate such a degradation in the generalization performance of a DNN: transductive learning and regularization. In transductive learning, a DNN is pre-trained with the training dataset and subsequently refined in the target dataset [14]–[16]. In a prior work, a CNN was pre-trained using chest X-ray images associated with adults, and then was refined using chest X-ray images associated with pediatric population for pediatric pneumonia diagnosis [17]. In another prior work, a CNN was pre-trained using digital mammography images, and then was refined using digital tomosynthesis images for breast cancer diagnosis [18]. Hence, transductive learning requires two training phases and labeled target dataset. In regularization, a DNN is trained with a regularization penalty imposed on the loss function to bias and generalize the DNN [19]. To properly bias the DNN to maximize its generalization capability, the regularization penalty must be judiciously formulated by considering the intended task and the characteristics of the dataset. Otherwise, the DNN may still be overfitted, or it may be underfitted and generalize too broadly, ultimately leading to poor performance in both training and target datasets. Regularization-based generalization has been pursued in various applications [20], [21]. For instance, a prior work on fault diagnosis of rotating machinery regularized the discriminant structure of a DNN to extract robust latent features generalizable to unseen domains [22]. In PAD diagnosis based on PWA, the morphology of arterial pulse signals (which are presented to the DNN as inputs) are influenced by PAD as well as other confounding factors, e.g., anatomical (e.g., height of a subject) and physiological (e.g., arterial biomechanical properties of a subject) characteristics of individual patients. A unique nature of these confounding factors is that they are continuous rather than discrete. Hence, an ideal regularization penalty for training a DNN suited to PAD diagnosis based on a scarce dataset must facilitate the generalization of the DNN across a wide range of continuous disturbances. In this work, we propose a novel regularization approach that can address such a challenge.

**FIGURE 1.**
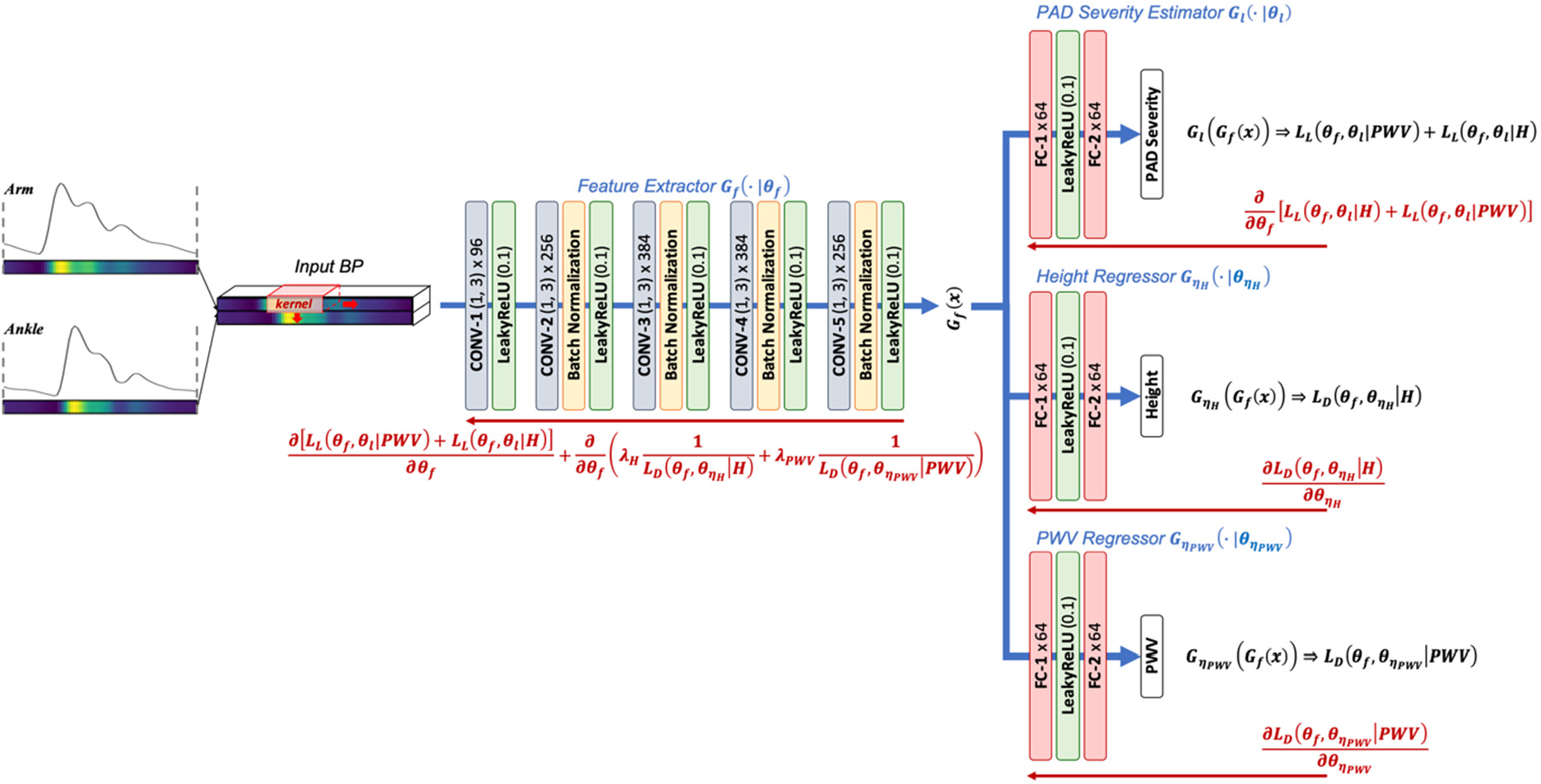
A CNN architecture for peripheral artery occlusive disease (PAD) diagnosis via CNN-based PWA.

Our regularization approach is inspired by the state-of-the-art domain-adversarial learning. In domain-adversarial learning, a DNN is trained to exploit the features relevant to the task but independent of the domain via domain-adversarial training [13], [23]. This is accomplished by defining the shift between a source domain (*D*_*S*_; training dataset) and a target domain (*D*_*T*_; test dataset and datasets encountered in real application of the DNN) as the H-divergence and utilizing it in training a DNN: the H-divergence serves as a penalty term in training the feature extraction layer, where it is minimized to set the parameters in the feature extraction layer so that the consequent features are regularized and domain-independent [24], [25]. Our CPAR approach extends the conventional domain-adversarial training (applicable mostly to discrete and categorical domains) to continuously connected source and target domains. Consider a domain defined by a continuous parameter *P*:

(1) $$P≜\left\{p|\underline{p}\leq p\leq \bar{p}\right\}$$

where the lower bound $\underline{p}$ and the upper bound $\bar{p}$ specify the range of *p*. For a given sample *x* in the domain associated with *p*_*x*_ ∈ *P*, we define the source and target domains as follows:

(2) $$D_{S}(p)=\left\{x|d\left(p,p_{x}\right)\leq \varepsilon \right\}\;D_{T}(p)=\left\{x|d\left(p,p_{x}\right)>\varepsilon \right\}$$

where *d* (*x*, *y*) ≜ tanh |*x* − *y*| is a distance measure between *x* and *y*, and *ε* > 0. Let the mapping in a DNN associated with the feature extraction layer, label prediction layer, and domain regression layer as *G*_*f*_ (*x*), *G*_*l*_ (*G*_*f*_ (*x*)), and *G*_*η*_ (*G*_*f*_ (*x*)), respectively (Figure 1). Denoting the set of latent features derived from the samples in the source and target domains pertaining to *p* ∈ *P* as follows:

(3) $$F_{S}(p)=\left\{G_{f}(x)|x\in D_{S}(p)\right\}\;F_{T}(p)=\left\{G_{f}(x)|x\in D_{T}(p)\right\}$$

the effective H-divergence $d_{H}^{eff}(P)$ between *F*_*S*_ (*p*) and *F*_*T*_ (*p*) across the entire (source and target) domain can be derived by integrating the H-idvergence $d_{H}\left(F_{S}(p),F_{T}(p)\right)$ associated with a specific value of *p* over all possible values of *p* in the domain. Given *n*_*S*_ (*p*) the number of samples from the source domain and *n*_*T*_ (*p*) the number of samples from the target domain, $d_{H}\left(F_{S}(p),F_{T}(p)\right)$ can be computed empirically as follows [13]:

(4) $$d_{H}\left(F_{S}(p),F_{T}(p)\right)≜2\left\{1-\left[\frac{1}{n_{S}(p)}\sum_{i=1}^{n_{S}(p)}I\left[\eta \left(G_{f}\left(x_{i}\right)=0|p\right)\right]+\frac{1}{n_{T}(p)}\sum_{j=1}^{n_{T}(p)}I\left[\eta \left(G_{f}\left(x_{j}\right)=1|p\right)\right]\right]\right\}$$

where *η*(·| *p*) is a binary classifier for source vs target domains in the hypothesis class H(p) associated with *p*, while *I* [·] is an indicator (i.e., *I* = 1 if the predicate is true and *I* = 0 otherwise). Then, $d_{H}^{eff}(P)$ is given by:

(5) $$d_{H}^{eff}(P)=\sum_{p}d_{H}\left(F_{S}(p),F_{T}(p)\right)$$

where the summation is an approximation to the integration over *p*. Then, it is possible to regularize the latent features against *P* (i.e., make them independent of *P*) by training a DNN so that $d_{H}^{eff}(P)$ is minimized, which allows the DNN to robustly perform the intended labeling task both in the source and target domains [13]. Inspired by Eq. (5), we conceive the following loss function for domain regression:

(6) $$L_{D}\left(\theta_{f},\theta_{\eta }|P\right)=\sum_{p}\left[\frac{1}{n_{S}(p)+n_{T}(p)}\sum_{k=1}^{n_{S}(p)+n_{T}(p)}L_{D}\left(x_{k},\theta_{f},\theta_{\eta }|p\right)\right]\;=\sum_{p}\left[\frac{1}{n_{S}(p)+n_{T}(p)}\sum_{k=1}^{n_{S}(p)+n_{T}(p)}\times \left\{\left(1-e\left(p,p_{x_{k}}\right)\right)\text{log}\frac{1}{d\left(p_{x_{k}},G_{\eta }\left(G_{f}\left(x_{k}\right)\right)\right)}+e\left(p,p_{x_{k}}\right)\text{log}\frac{1}{1-d\left(p_{x_{k}},G_{\eta }\left(G_{f}\left(x_{k}\right)\right)\right)}\right\}\right]$$

where $e\left(p,p_{x_{k}}\right)≜\{\begin{array}{l} 1,d\left(p,p_{x_{k}}\right)\leq \varepsilon \\ 0,d\left(p,p_{x_{k}}\right)>\varepsilon \end{array}$, and *Gη* (*Gf* (*x*_*k*_)) is the domain regressed by the DNN when *x*_*k*_ is inputted. We employ the standard 2-norm-based loss function for label prediction:

(7) $$L_{L}\left(\theta_{f},\theta_{l}|P\right)=\sum_{p}\left[\frac{1}{n_{S}(p)+n_{T}(p)}\sum_{k=1}^{n_{S}(p)+n_{T}(p)}L_{L}\left(x_{k},\theta_{f},\theta_{l}|p\right)\right]\;=\sum_{p}\left[\frac{1}{n_{S}(p)+n_{T}(p)}\sum_{k=1}^{n_{S}(p)+n_{T}(p)}{\left(y_{k}-G_{l}\left(G_{f}\left(x_{k}\right)\right)\right)}^{2}\right]$$

where *y*_*k*_ is the label associated with *x*_*k*_. Then, training a DNN with CPAR boils down to solving the following set of optimization problems:

(8) $$\theta_{l}^{*}=\text{arg }\underset{\theta_{l}}{\text{min }}L_{L}\left(\theta_{f},\theta_{l}|P\right)\;\theta_{\eta }^{*}=\text{arg }\underset{\theta_{\eta }}{\text{min }}L_{D}\left(\theta_{f},\theta_{\eta }|P\right)\;\theta_{f}^{*}=\text{arg }\underset{\theta_{f}}{\text{min }}L_{L}\left(\theta_{f},\theta_{l}|P\right)+\lambda \frac{1}{L_{D}\left(\theta_{f},\theta_{\eta }|P\right)}$$

where *λ* > 0 is the regularization weight. It is noted that the way *L*_*D*_ (*θ*_*f*_, *θ*_*n*_ | *P*) is incorporated into the training of *θ*_*η*_ and *θ*_*f*_ are distinct in Eq. (8). Indeed, given that *L*_*D*_ (*θ*_*f*_, *θ*_*η*_ | *P*) > 0 and its value increases as domain regression fails, *θ*_*η*_ can be optimized by minimizing *L*_*D*_ (*θ*_*f*_, *θ*_*n*_ | *P*). In contrast, if *L*_*D*_ (*θ*_*f*_*, θ*_*η*_ | *P*) is incorporated into the training of *θ*_*f*_ in the classical way (i.e., optimizing *θ*_*f*_ by minimizing *L*_*L*_ (*θ*_*f*_ *,θ*_*l*_ | *P*) − *λL*_*D*_ (*θ*_*f*_*, θ*_*η*_ | *P*)), then the cost function may behave poorly: (i) the loss function is no longer lower-bounded since *L*_*D*_ (*θ*_*f*_*, θ*_*η*_ | *P*) can grow indefinitely, and (ii) CPAR cannot be effectively enforced when *L*_*D*_ (*θ*_*f*_*, θ*_*η*_ | *P*) ≈ 0 because the contribution of *L*_*D*_ (*θ*_*f*_*, θ*_*η*_ | *P*) to the gradient becomes negligible. Hence, our choice of the loss function for training of *θ*_*f*_ has several advantages: (i) it is positive and lower-bounded at zero; (ii) it is large when *L*_*D*_ (*θ*_*f*_*, θ*_*η*_ | *P*) is small, which promotes regularization by CPAR; and (iii) it gets dominated by *L*_*L*_ (*θ*_*f*_ *,θ*_*l*_ | *P*) as *L*_*D*_ (*θ*_*f*_*, θ*_*η*_ | *P*) grows indefinitely, which promotes training of *θ*_*η*_ to improve the domain regression layer while minimizing the CPAR action.

It is worth mentioning that our CPAR approach can be readily extended to simultaneously deal with multiple domains pertaining to more than one continuous parameters $P_{i}≜\left\{p_{i}|\underline{p_{i}}\leq p_{i}\leq {\bar{p}}_{i}\right\}$, simply by connecting multiple domain regression layers to the feature extraction layer (see Figure 1 for a CNN with two domain regression layers). In this case, the loss functions in Eq. (8) are extended to the following, where *N*_*P*_ is the number of domain parameters:

(9) $$\theta_{l}^{*}=\text{arg }\underset{\theta_{l}}{\text{min }}\sum_{P_{i}}L_{L}\left(\theta_{f},\theta_{l}|P_{i}\right)\;\theta_{\eta_{i}}^{*}=\text{arg }\underset{\theta_{\eta_{i}}}{\text{min }}L_{D}\left(\theta_{f},\theta_{\eta_{i}}|P_{i}\right),\;i=1,\cdots N_{P}\;\theta_{f}^{*}=\text{arg }\underset{\theta_{f}}{\text{min }}\sum_{P_{i}}L_{L}\left(\theta_{f},\theta_{l}|P_{i}\right)+\lambda_{i}\frac{1}{L_{D}\left(\theta_{f},\theta_{\eta_{i}}|P_{i}\right)}$$

### B. PAD DIAGNOSIS BASED ON CONTINUOUS PROPERTY-ADVERSARIAL REGULARIZATION

We applied the CPAR approach in Section II.A to the development of a CNN for PAD diagnosis under dataset limitation constraints. As shown in Figure 1, the CNN architecture consists of a modified AlexNet [26] structure (whose efficacy in PAD diagnosis was demonstrated in our prior work [11]) combined with two domain regression layers. The CNN receives arterial pulse wave signals at an arm (brachial artery) and an ankle (tibial artery) as inputs to perform PWA and diagnose PAD. The label prediction layer predicts PAD severity. The domain regression layers are used in the training phase to maximize the extraction of features independent of two key anatomical and physiological characteristics affecting the morphology of arterial pulse wave signals and thus the efficacy of PAD diagnosis: height and arterial stiffness (using PWV as a surrogate measure). Details follow.

#### 1) VIRTUAL DATASET CREATION

**FIGURE 2.**
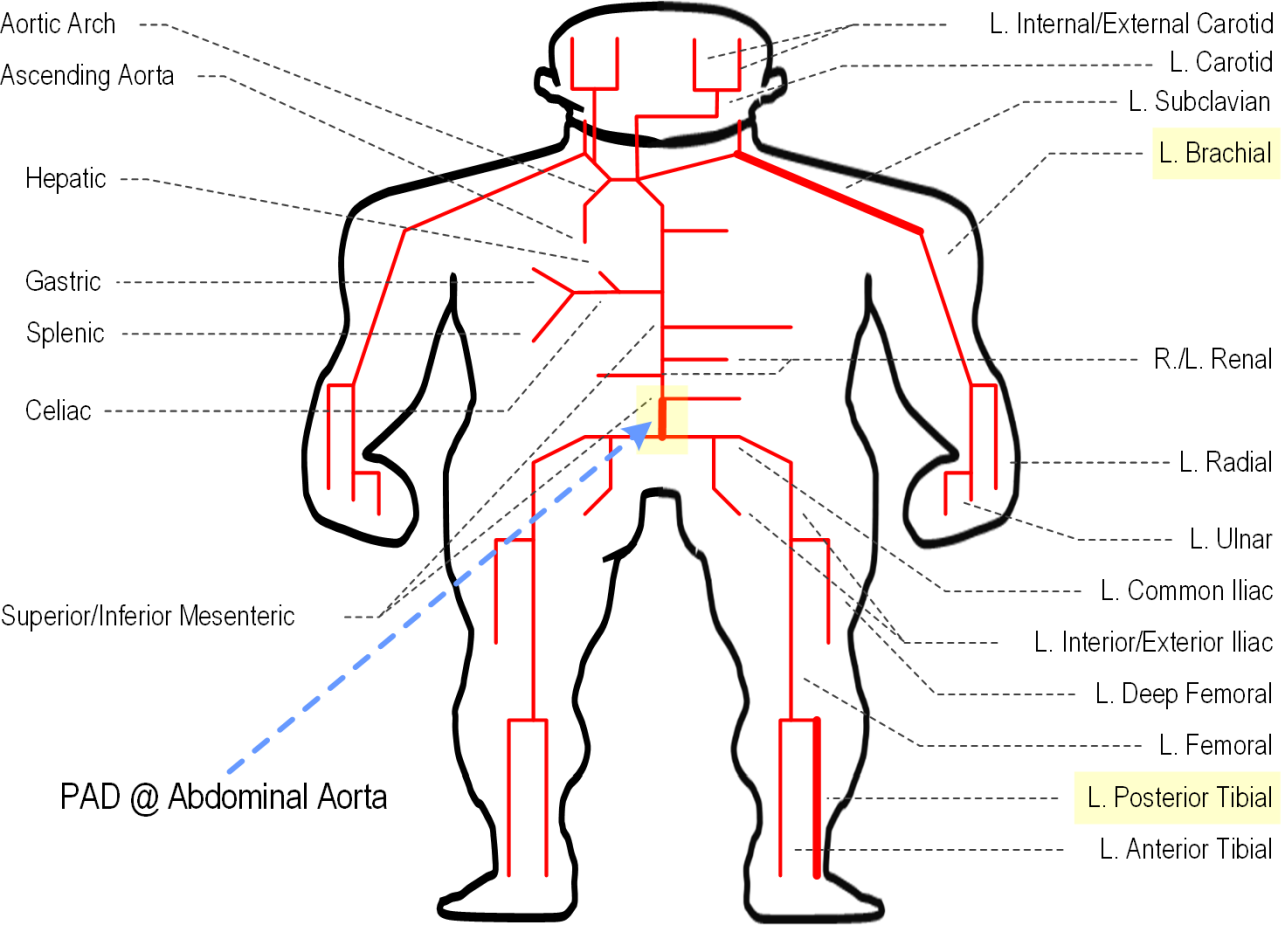
A lumped-parameter mathematical model of human arterial tree based on the transmission line theory.

We created virtual datasets for training and testing of a CNN for PAD diagnosis using an established lumped-parameter mathematical model of human arterial tree based on the transmission line (TL) theory [27] (Figure 2). The mathematical model consists of 55 TLs, each of which represents a segment of arterial tree characterized by segment-specific viscous, elastic, and inertial properties. Full details of the mathematical model is provided in He *et al.* [27]. This mathematical model was validated using physiological measurements as well as the results of other studies, and was used in the study of arterial stenosis and arterial viscoelasticity [10], [28], [29] as well as PAD diagnosis [10], [11].

We created virtual datasets by perturbing a few anatomical and arterial mechanical parameters in the lumped-parameter mathematical model from their respective nominal values. We specifically varied (i) height, (ii) stiffness, diameter, and thickness of all the arteries, and (iii) resistances associated with terminal arteries. Then, we simulated the mathematical model equipped with these parameters to obtain a wide range of virtual brachial and tibial artery BP signals. We considered both inter-individual variability (IIV) and intra-individual uncertainty in perturbing these parameters. We considered the IIV in height in the range of 162–198cm (+/−10% perturbation with respect to nominal value). We considered the IIV in arterial diameter, thickness, and peripheral load resistance parameters of +/−20% perturbation around their respective nominal values. We considered the IIV in arterial stiffness to replicate the range of PWV observed in adults of 50–60 years in age in hypertension cohort (systolic BP>160mmHg and diastolic BP>100mmHg; PWV 4.8–15.1m/s) [30]. In combination with the IIV associated with arterial diameter, thickness, and peripheral load resistances, PWV in the range of 4.4–15.8m/s was obtained by perturbing arterial stiffness over −20%–400% of its nominal value. To create multiple arterial pulse signal samples from each virtual patient obtained with the IIVs described above, we considered intra-individual uncertainty in the form of lognormal distributions associated with the five anatomical (i.e., height) and arterial mechanical (i.e., arterial stiffness, diameter, thickness, and peripheral load resistance) parameters, with subject-specific values (nominal plus IIV) as mean and coefficient of variation of 0.01.

We considered PAD at the abdominal aorta (which is one of the most common PAD locations). To simulate PAD with increasing severity, we decreased the diameter of the abdominal aorta from its nominal value. We defined the PAD severity as the degree of occlusion: 0% when normal, and 100% when fully occluded.

To create training dataset, we randomly sampled 32 virtual patients based on the IIV described above and a randomly chosen PAD severity (from within 0%–80%). From each virtual patient, we created 1,000 random samples subject to intra-individual anatomical and physiological variability by applying the intra-individual uncertainty described above to the five anatomical and arterial mechanical parameters. Then, we repeatedly simulated the mathematical model 32,000 times by characterizing it by each of the 32,000 random parameter samples and the corresponding PAD severity levels to create brachial and tibial BP pulse signals. The resulting 32,000 arterial BP pulse signals pertaining to the 32 virtual patients along with the PAD severity, height, and PWV associated with these arterial BP pulse signals formed the training dataset.

To create test dataset, we sampled a large number of virtual patients by widely changing the IIV corresponding to the five anatomical and arterial mechanical parameters by 5% increments (5 (height: −10%, −5%, 0%, +5%, +10%) × 9 (artery diameter: −20%, −15%, …+15%, +20%) × 9 (artery thickness: −20%, −15%, …+15%, +20%) × 9 (peripheral load resistance: −20%, −15%, …+15%, +20%) × 77 (artery stiffness: −20%, −15%, …+395%, +400%) = 280,665) and PAD severity by 10% increment (from within 0%–80%). From each virtual patient at a specific PAD severity level, we created 10 random samples subject to intra-individual anatomical and physiological variability by applying the intra-individual uncertainty described above to the five anatomical and arterial mechanical parameters. Then, we repeatedly simulated the mathematical model 25,259,850 (=280,665 × 9 PAD severity levels) times by characterizing it by each of the 2,806,650 random parameter samples and all the PAD severity levels (0%–80%) to create brachial and tibial BP pulse signals. The resulting 25,259,850 arterial BP pulse signals pertaining to the 280,665 virtual patients along with the PAD severity associated with these arterial BP pulse signals formed the test dataset. The rationale behind creating a large-size test data was to extensively evaluate the efficacy of the proposed PAD diagnosis approach via CNN-based PWA.

#### 2) TRAINING, TESTING, AND ANALYSIS OF CNN FOR PAD DIAGNOSIS

The morphology of arterial pulse wave signals are influenced by confounding factors arising from anatomical and arterial mechanical characteristics as well as PAD severity. According to the Moens-Korteweg equation [12], arterial stiffness, diameter, and thickness alter the shape of arterial pulse wave signals by changing PWV (which impacts the timings with which forward and backward traveling pulse waves are superimposed [12]). In addition, height also alters the shape of arterial pulse wave signals by changing the length of pulse wave travel paths. To achieve robust PAD diagnosis accuracy irrespective of these disturbances, we applied the CPAR approach to the training of a CNN for PAD diagnosis via PWA. Our prior work suggested that height and arterial stiffness exerts large influence on the morphology of arterial pulse wave signals among all the disturbances due to anatomical and arterial mechanical characteristics [11]. Hence, we included two domain regression layers in the CNN as shown in Figure 1, and used the CPAR approach so that the features extracted from the brachial and tibial BP pulse signals and inputted to the label prediction layer for PAD diagnosis do not include those indicative of height and arterial stiffness.

Considering that direct non-invasive measurement of arterial stiffness is extremely challenging if not impossible, we employed PWV as a surrogate of arterial stiffness. Although PWV is dependent on arterial diameter and thickness as well as arterial stiffness [12], the observation from our prior work showed that the effect of arterial stiffness is predominant in altering the shape of arterial BP pulse signals among all the arterial mechanical properties [11], which suggests that PWV may serve as a credible surrogate of arterial stiffness.

We evaluated the efficacy of our CPAR-trained CNN-based PWA approach to PAD diagnosis for its ability to detect PAD and assess PAD severity by training and testing the CNN using the training and test datasets outlined in Section II.A.1. To robustly evaluate our approach, we repeatedly created the training and test datasets 10 times, evaluated the performance of the CNN, and reported the efficacy of our approach in terms of the aggregated performance of the CNN obtained from the 10 tests. In training the CNN, we used the ADAM optimizer with *α* = 0.5, *β* = 0.999, the learning rate of 0.0001, and the regularization weight (*λ*) of 0.002.

Our performance metrics included (i) sensitivity, specificity, accuracy, and area under the ROC curve (AUC) as the measures of PAD detection and (ii) r^2^ value and root-mean-squared error (RMSE) between actual vs CNN-predicted PAD severity as the measures of PAD severity assessment, all derived using the test dataset. We evaluated the detection accuracy of our CPAR-trained CNN-based PWA approach to PAD diagnosis against a number of PAD severity values as the PAD labeling threshold. To examine the advantage of PWA over conventional ABI technique, we compared the performance metrics associated with PWA and ABI. To enable objective comparison, we mapped ABI value to PAD severity via a pre-calibrated polynomial regression model relating ABI to PAD severity (which was derived based on the virtual patient characterized by the nominal anatomical and physiological parameter values).

To scrutinize the role of CPAR in promoting the use of features independent of domain disturbances, we compared the PAD diagnosis efficacy of the CNN trained with (i) CPAR, (ii) conventional learning without CPAR (in which no domain-adversarial regularization is enforced), and (iii) multi-task learning (which, as opposed to CPAR, fosters the exploitation of the features commonly useful for PAD diagnosis and domain regression) using the test dataset. In addition, we investigated the performance of our CNN-based PWA approach to PAD diagnosis with respect to the extent to which CPAR is applied, by comparing the CNN trained with CPAR applied to height only, PWV only, and both height and PWV using the test dataset. Finally, to assess the validity of PWV as a credible surrogate of arterial stiffness, we also trained the CNN with arterial stiffness used for domain regularization, and compared its performance with the CNN with PWV used for domain regularization using the test dataset.

## III. RESULTS

**FIGURE 3.**
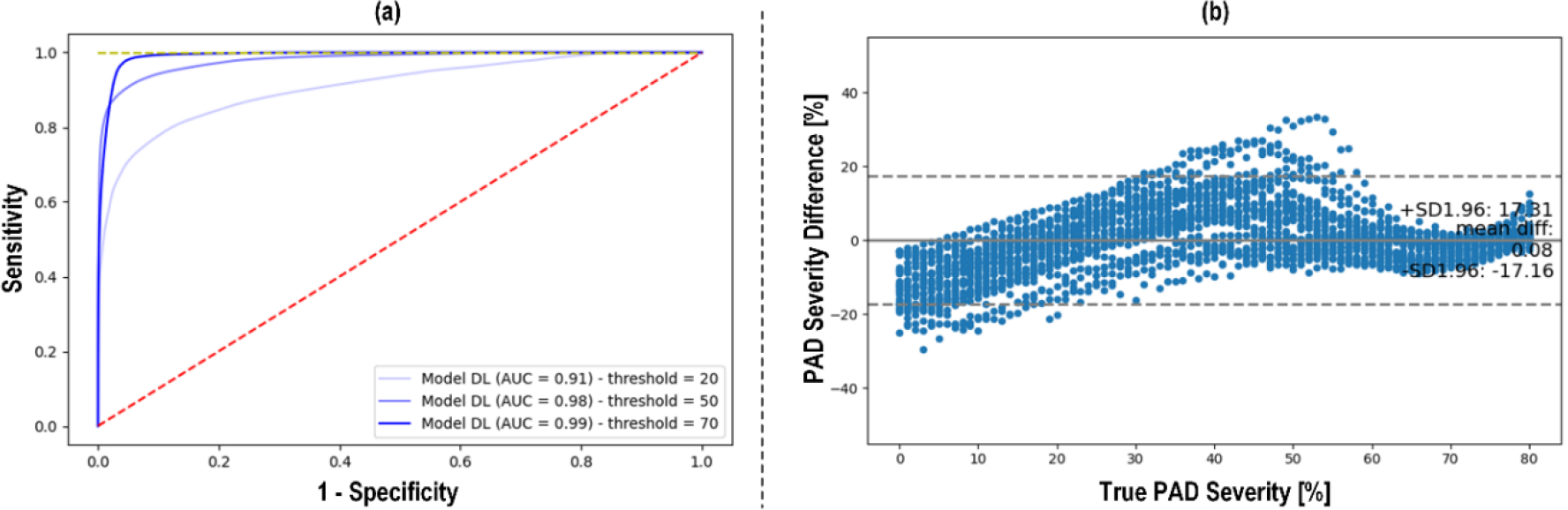
Efficacy of CNN-based PWA approach to PAD diagnosis. (a) Receiver operating characteristic (ROC) curve. (b) Bland-Altman plot between actual vs predicted PAD severity.

**FIGURE 4.**
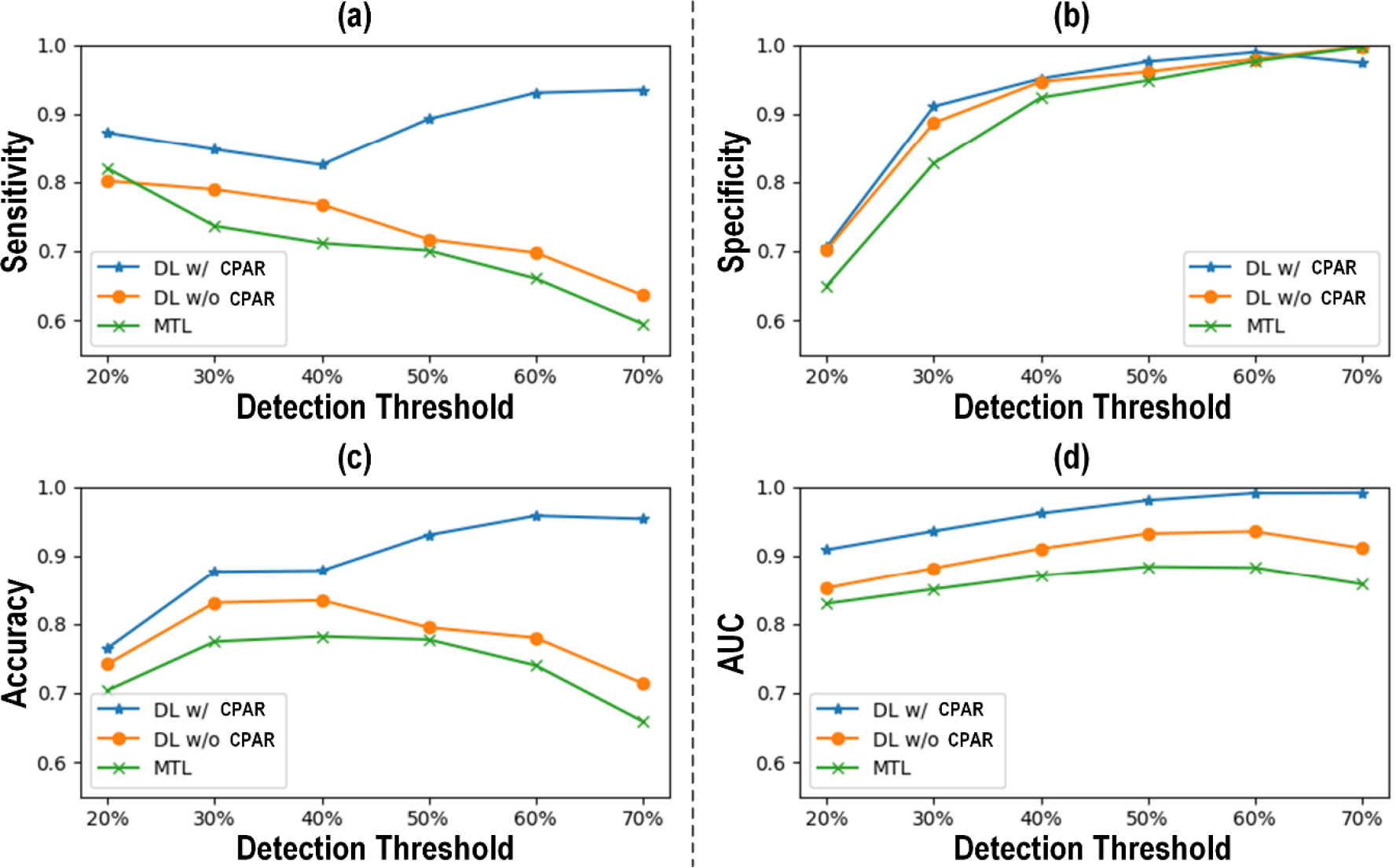
PAD detection accuracy associated with the CNN trained with CPAR, conventional learning without CPAR, and multi-task learning. (a) Sensitivity. (b) Specificity. (c) Accuracy. (d) AUC.

**FIGURE 5.**
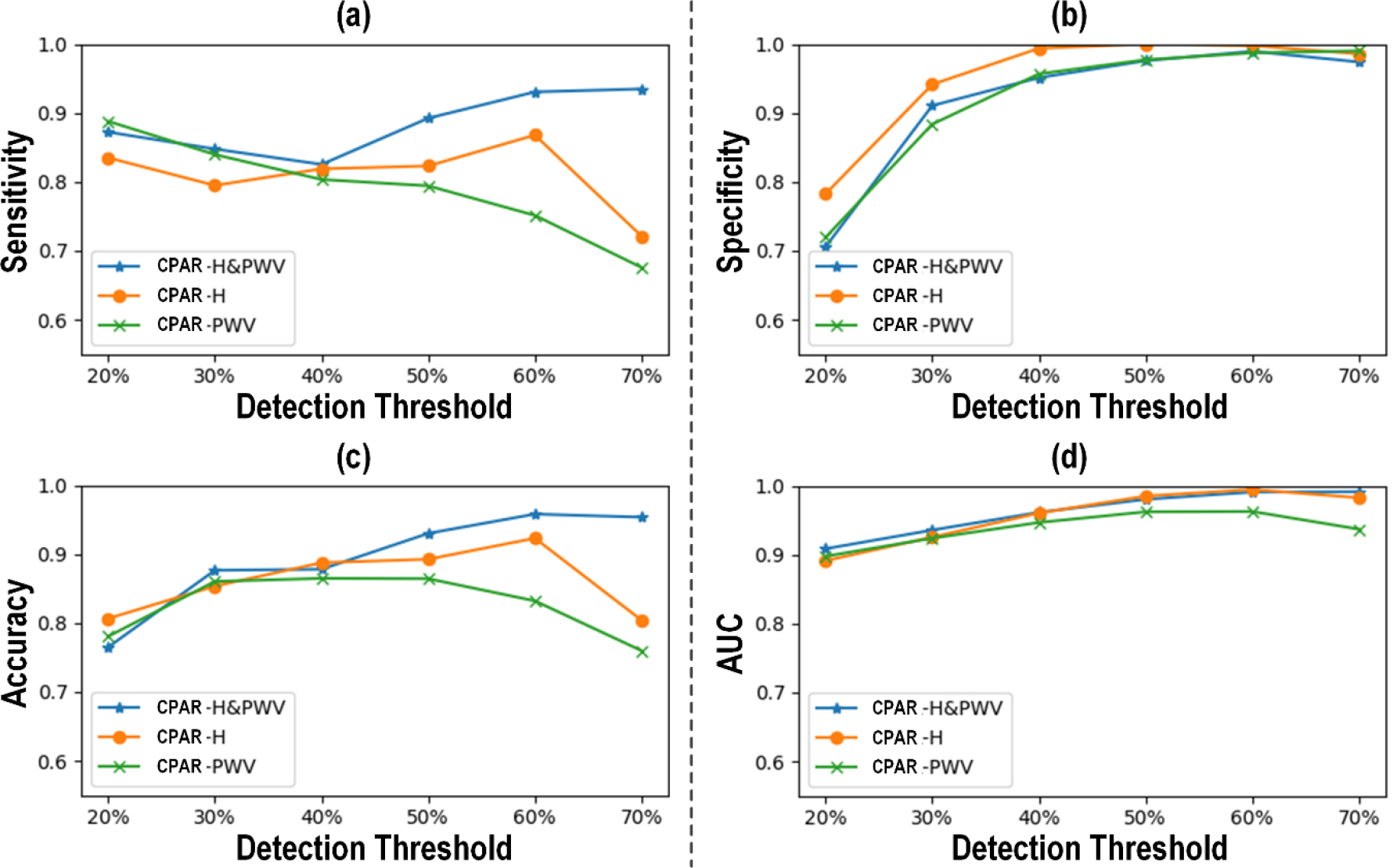
PAD detection accuracy of the CNN trained with CPAR applied to height only (H), PWV only (PWV), and both height and PWV (H&PWV). (a) Sensitivity. (b) Specificity. (c) Accuracy. (d) AUC.

**FIGURE 6.**
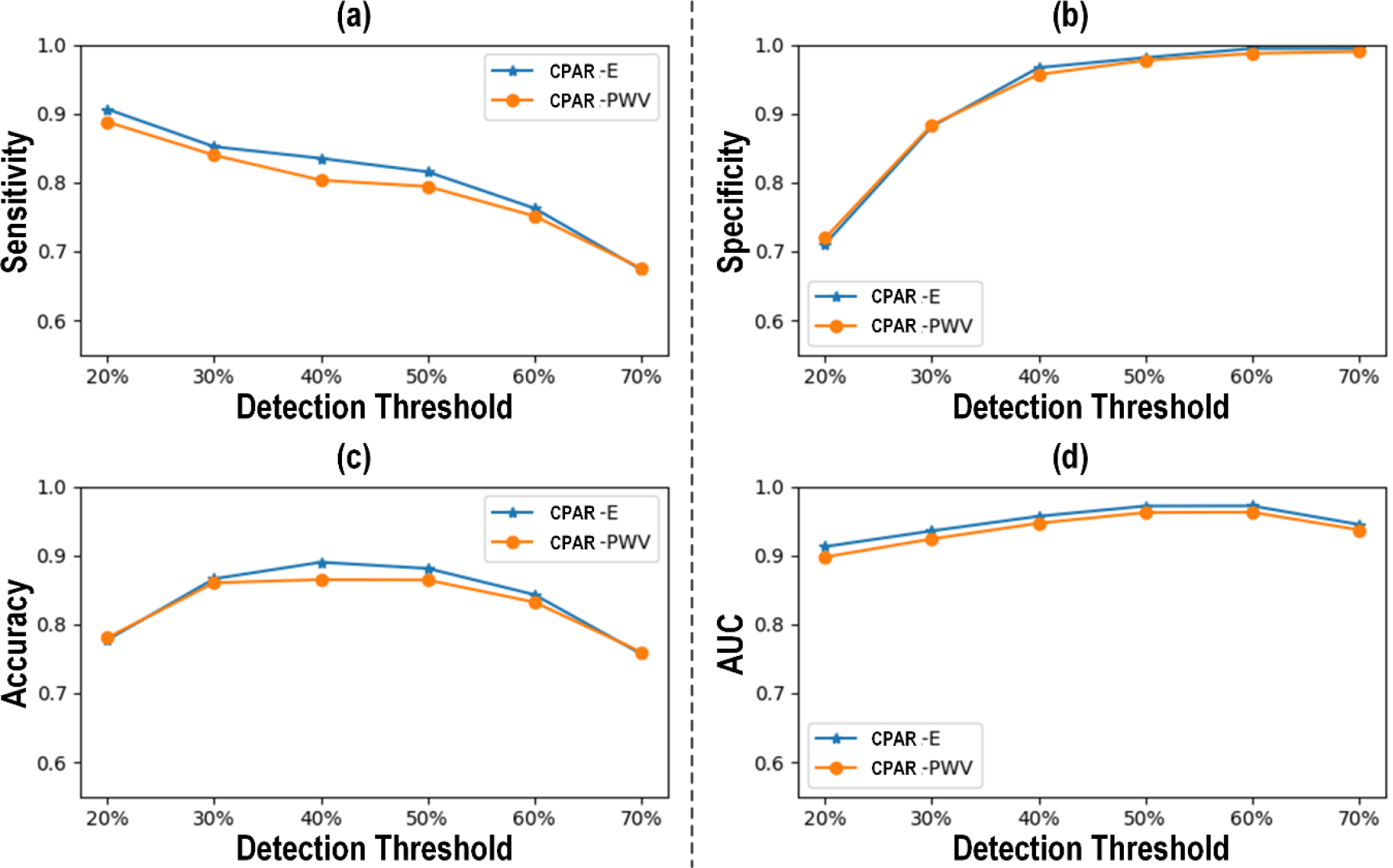
PAD detection accuracy of the CNN trained with PWV (PWV) and arterial stiffness (E) used for domain regularization.

Figure 3 shows (a) the receiver operating characteristic (ROC) curve and (b) the Bland-Altman plot between actual vs predicted PAD severity associated with our CNN-based PWA approach to PAD diagnosis. Figure 4 compares the PAD detection accuracy associated with the CNN trained with (i) CPAR, (ii) conventional learning without CPAR, and (iii) multi-task learning. Figure 5 compares the PAD detection accuracy of the CNN trained with CPAR applied to (i) height only, (ii) PWV only, and (iii) both height and PWV. Figure 6 compares the PAD detection accuracy of the CNN trained with (i) PWV and (ii) arterial stiffness used for domain regularization, respectively. TABLE 1 summarizes the PAD severity assessment accuracy associated with the ABI technique as well as CNN trained with (i) CPAR, (ii) conventional learning without CPAR, and (iii) multi-task learning.

## IV. DISCUSSION

Current PAD diagnosis requires imaging-based methods not suited to convenient screening and surveillance purposes. In addition, the ABI technique suffers from poor diagnostic accuracy. DL-based PWA has the potential to make a leap in PAD diagnosis by virtue of its ability to automatically exploit the features indicative of PAD from readily measurable arterial pulse wave signals. However, the morphology of arterial pulse wave signals are influenced by anatomical and arterial mechanical characteristics as well as PAD. If combined with only scarce dataset from a small number of patients (who possibly encompass only a narrow range of anatomical and physiological characteristics) available for training of CNN, these confounding factors can deteriorate the training of CNN by increasing the risk of the CNN overfitted only to the individuals in the training dataset while deteriorating its generalizability to unseen individuals who may exhibit more diverse anatomical and physiological characteristics beyond the training dataset. To enable robust diagnosis of PAD against these challenges, we developed a novel CPAR approach and applied it to the development of a CNN-based PWA approach to PAD diagnosis. Below we demonstrate the potential of CPAR in enabling robust PAD diagnosis against confounding anatomical and physiological disturbances.

### A. EFFICACY OF CPAR

The CNN trained with CPAR exhibited adequate PAD detection and assessment accuracy (Figure 3). It showed AUC consistently higher than 0.9 across all the detection thresholds considered in this work, and achieved nearly unbiased severity assessment (bias <0.1%).

Comparing the CNN trained with CPAR against the CNN trained without CPAR and another CNN trained with multi-task learning ascertained the role of CPAR in robustifying the CNN against anatomical and arterial mechanical disturbances (Figure 4 and TABLE 1). The CNN trained with CPAR was superior to the CNN trained without CPAR and the CNN trained with multi-task learning in both detection and severity assessment aspects. Specifically, the CPAR-trained CNN exhibited superior sensitivity while maintaining specificity comparable to its competitors (by virtue of its unbiased estimation of PAD severity, in contrast to its competitors in which PAD severity was underestimated on the average), thereby achieving superior accuracy and AUC characteristics. It is noted that the CNN trained without CPAR was superior to the CNN trained with multi-task learning. Noting that (i) the CNN trained without CPAR does not enforce any action against domain disturbances, and that (ii) multi-task learning fosters the extraction of latent features indicative of domain disturbances in performing PAD diagnosis, the results suggest that regularizing the latent features with CPAR can improve the performance of the intended task susceptible to domain-induced disturbances by making them independent of (or at least less dependent on) domain disturbances.

The advantage of CPAR may also be ascertained from the fact that the efficacy of CNN-based PWA approach to PAD diagnosis was improved as CPAR was applied simultaneously to multiple domain-induced disturbances than to one domain disturbance at a time (Figure 5). The CNN trained with CPAR to regularize against height and PWV exhibited consistently higher sensitivity than those trained to regularize only against height or PWV, despite slightly lower specificity than the height-regularized CNN (which, again, appears to be due to its unbiased estimation of PAD severity, in contrast to its competitorsinwhichPADseveritywasunderestimatedonthe average). The CNN regularized against both height and PWV was persistently superior to the one regularized only against PWV in terms of accuracy and AUC. In terms of accuracy and AUC characteristics, the CNN regularized against both height and PWV was not consistently superior to its competitor regularized only against height. However, considering all the performance aspects (sensitivity in particular), we contend that the CNN regularized against both height and PWV may still be superior to the ones regularized only against height or PWV.

All in all, our results strongly demonstrate the potential of CPAR in realizing CNN-based PWA approach to PAD diagnosis. CPAR can derive a CNN robust against anatomical and arterial mechanical properties altering the morphology of arterial BP pulse signals, by virtue of its ability to foster the exploitation of latent features independent of the domain-induced disturbances in performing PAD diagnosis task even when the dataset available for training is scarce and non-longitudinal.

### B. POTENTIAL FOR CLINICAL APPLICABILITY

A few critical questions arise regarding the potential of the CNN-based PWA approach to PAD diagnosis for real-world clinical application: (i) is it superior to the current state-of-the-art; and (ii) does the use of PWV as a surrogate of arterial stiffness drastically impact the PAD diagnosis efficacy?

The CNN-based PWA approach to PAD diagnosis was much superior to the ABI technique in terms of both detection and severity assessment (TABLE 1). One implication is that the exploitation of the entire arterial pulse signals is more efficacious than the use of isolated fiducial points therein in diagnosing PAD. This implication justifies the complexity in the measurement and analytics required for PWA. Another implication is that the CNN-based PWA approach can leverage CPAR to improve its robustness against domain-induced disturbances due to the anatomical and arterial mechanical characteristics, whereas the ABI technique is inevitably prone to those disturbances.

The use of PWV instead of (practically unmeasurable) arterial stiffness did not largely compromise the efficacy of CNN-based PAD diagnosis (Figure 6). Indeed, the former exhibited only a marginal drop in the sensitivity, accuracy, and AUC characteristics relative to the latter, while the specificity was more or less maintained. This implies that, although PWV is not a perfect surrogate of arterial stiffness due to its reliance on other arterial mechanical properties, it may still be a viable choice to enable real-world implementation of the CNN-based PWA approach to PAD diagnosis in conjunction with CPAR-augmented learning with regularization.

All in all, our results suggest that CPAR-trained CNN-based PWA approach to PAD diagnosis may open up unprecedented opportunities for improving the convenience and accuracy of PAD screening and surveillance.

## V. CONCLUSION

In this paper, we proposed CPAR, an enabling methodology for robust training and generalization of a DNN to detect and assess the severity of PAD against continuous property-induced disturbances arising from anatomical and arterial mechanical characteristics. The results demonstrated the ability of the CPAR to promote the extraction of latent features independent of domain disturbances, thereby robustifying the performance of PAD diagnosis against a wide range of IIV in height and arterial stiffness. Given the proof-of-concept nature of this work based on virtual in silico dataset, future work must investigate the in vivo efficacy of the proposed PAD diagnosis approach based on CPAR-trained CNN. In addition, it may be worth investigating the potential of CPAR-trained deep learning in the diagnosis of diseases other than PAD.

## Figures and Tables

**TABLE 1.** PAD severity assessment accuracy associated with the ABI technique as well as CNN trained with (i) CPAR, (ii) conventional learning without CPAR, and (iii) multi-task learning.

	ABI	DL (NO CPAR)	DL (CPAR)	MULTI-TASK LEARNING
RMSE [%]	23.0	15.0	9.5	16.9
r^2^ Value	0.034	0.588	0.834	0.478
